# Editorial: Demyelinating neurological syndromes: The role of autoimmunity

**DOI:** 10.3389/fneur.2023.1178980

**Published:** 2023-03-24

**Authors:** Maria Eleftheria Evangelopoulos, Robert Hoepner, Clio Mavragani

**Affiliations:** ^1^1st Department of Neurology, Eginition Hospital, National and Kapodistrian University of Athens, Athens, Greece; ^2^Department of Neurology, Inselspital, Bern University Hospital, University of Bern, Bern, Switzerland; ^3^Greece and Joint Academic Rheumatology Program, Departments of Physiology and Pathophysiology, School of Medicine, National and Kapodistrian University of Athens, Athens, Greece

**Keywords:** demyelinating diseases, autoimmunity, multiple sclerosis (MS), systemic lupus erythematosus (SLE), neuromyelitis optica (NMO), Sjogren's syndrome, type I interferon (IFN)

Demyelination of the central nervous system (CNS) is a clinical entity with diverse etiology, often challenging for the practicing neurologist in both diagnostic and therapeutic terms. While multiple sclerosis (MS) spectrum disorders are the commonest demyelinating CNS diseases, important mimickers should be always carefully excluded prior to final diagnosis. For instance, systemic autoimmune disorders such as systemic lupus erythematosus (SLE) and Sjogren's syndrome (SS) can present with lesions highly reminiscent of MS. In these cases, distinction between those entities can be remarkably complex, especially in the absence of a known history of systemic autoimmune disease or florid systemic autoimmune features. In the current Research Topic, pathogenetic mechanisms, clinical spectrum, as well as diagnostic and therapeutic interventions in demyelinating syndromes of various autoimmune etiology are discussed.

In a recent study by Nikolopoulos et al., including 707 patients with SLE, 3.7% presented with CNS demyelinating syndromes. Approximately half of them displayed demyelinating features attributed primarily to lupus with the other half fulfilling criteria for MS, characterized as SLE/MS overlap. While neurological and rheumatological manifestations did not significantly differ between groups, patients with SLE and demyelination had mostly mild manifestations beyond CNS (mainly affecting joints and skin), mild neurological disease activity and were less likely to display heightened IgG index, positive oligoclonal bands or infratentorial, periventricular and juxtacortical brain lesions (Nikolopoulos et al.).

In line with these findings, in a large cohort including patients presenting with undifferentiated demyelinating CNS disease, patients either fulfilling classification criteria for a well-defined systemic autoimmune disease or had autoimmune clinical and/or serological features had lower rates of infratentorial and callosal MRI lesions, CSF T2 oligoclonal bands, and IgG-index positivity compared to MS patients. These patients were older, more frequently females, with increased rates of hypertension/hyperlipidemia, family history of autoimmunity, cortical dysfunction, anti-nuclear antibody titers ≥1/320, anticardiolipin IgM positivity, and atypical for MS magnetic resonance imaging lesions. Of note patients fulfilling criteria for systemic autoimmune disease had significantly higher peripheral blood type I interferon IFN scores at baseline compared to patients with MS spectrum disorders (1).

Due to the small number of patients with Neuromyelitis optica spectrum disorders (NMOSD) included in the study it was not possible to detect heightened type I IFN scores in this patient population though recent data revealed that type I IFN activated microglia could be contributing to its pathogenesis (2).

Activation of IFN signaling pathways and shared genetic contributors as reviewed in the report by Wang et al. and Ghafouri-Fard et al. could account for the well-recognized coexistence between NMOSD and systemic autoimmune diseases such as SLE and Sjögren's syndrome.

Type I interferons (IFNs), the main antiviral body defense, displays immunomodulatory properties with IFN-β being a well-established therapeutic modality for MS. In the review by Raftopoulou et al. the key mechanisms of type I IFN production by CNS cellular populations as well as its local effects on the CNS are discussed. Moreover, the contributory role of type I IFNs in the pathogenesis of neuropsychiatric lupus erythematosus and type I interferonopathies is presented (Raftopoulou et al.).

Toll-like receptors (TLRs) which are key sensors of external or internal stimuli have been shown to be active participants in inflammatory and antiinflammatory responses and type I IFN production characterizing neuroimmune diseases with TLR signaling pathway being a potential therapeutic target (Li H. et al.).

While identification of primary triggers for immune activation is not clear and still unexplored, viruses such as Epstein Barr virus and cholesterol remnants, have been shown to lead to monocyte activation (Ding et al.; Li L. et al.).

In conclusion, it is increasingly recognized that immune pathway activation has a chief role in clinical and pathogenetic features of patients presenting with several neurological features. Further research is required to elucidate distinct pathogenetic pathways and establish tailored therapeutic approached.

## Author contributions

ME and CM contributed to the writing and revision of the manuscript. RH contributed to the revision of the manuscript. All authors contributed to the article and approved the submitted version.
